# Workforce analysis using data mining and linear regression to understand HIV/AIDS prevalence patterns

**DOI:** 10.1186/1478-4491-6-2

**Published:** 2008-01-31

**Authors:** Elizabeth A Madigan, Olivier Louis Curet, Miklos Zrinyi

**Affiliations:** 1 Frances Payne Bolton School of Nursing, Case Western Reserve, 10900, Euclid Ave., Cleveland OH 44106-4904, USA; 2 Frances Payne Bolton School of Nursing, Case Western Reserve University, 10900 Euclid Ave., Cleveland OH 44106-4904, USA; 3 World Health Organization, Geneva, Switzerland

## Abstract

**Background:**

The achievement of the Millennium Development Goals (MDGs) depends on sufficient supply of health workforce in each country. Although country-level data support this contention, it has been difficult to evaluate health workforce supply and MDG outcomes at the country level. The purpose of the study was to examine the association between the health workforce, particularly the nursing workforce, and the achievement of the MDGs, taking into account other factors known to influence health status, such as socioeconomic indicators.

**Methods:**

A merged data set that includes country-level MDG outcomes, workforce statistics, and general socioeconomic indicators was utilized for the present study. Data were obtained from the Global Human Resources for Health Atlas 2004, the WHO Statistical Information System (WHOSIS) 2000, UN Fund for Development and Population Assistance (UNFDPA) 2000, the International Council of Nurses "Nursing in the World", and the WHO/UNAIDS database.

**Results:**

The main factors in understanding HIV/AIDS prevalence rates are physician density followed by female literacy rates and nursing density in the country. Using general linear model approaches, increased physician and nurse density (number of physicians or nurses per population) was associated with lower adult HIV/AIDS prevalence rate, even when controlling for socioeconomic indicators.

**Conclusion:**

Increased nurse and physician density are associated with improved health outcomes, suggesting that countries aiming to attain the MDGs related to HIV/AIDS would do well to invest in their health workforce. Implications for international and country level policy are discussed.

## Background

The socio-political impact of HIV/AIDS is increasingly being identified as a global crisis, rather than only a health crisis. There are estimates that generations of economic development and progress will be woefully reduced by the pandemic and that the socio-political fall-out due to HIV/AIDS has only started. Complacency in the developed world is risky because of the global nature of the world economic markets and the inter-dependencies for trade and manufacturing that are complex and multi-national. Nonetheless, data regarding mortality, especially infant mortality, and life expectancy indicate marked disparities between rich and poor countries [1].

One of the United Nations Millennium Development Goals (MDGs) is to reverse the spread of HIV/AIDS by 2015. Providing safe and effective anti-retroviral medications to HIV positive patients in developing countries will help enhance positive health outcomes [2]. However, in addition to the provision of anti-retroviral medications, there are additional health system factors that may influence the achievement of the MDGs. One of these factors is the health care workforce itself. In the case of Antiretroviral Drugs (ARVs), the influence of the healthcare workforce is obvious. Healthcare workers are required to provide the medication and to teach patients about and monitor both side effects and effectiveness of the ARVs.

The WHO, in addition to focusing on basic public health and epidemiology, also focuses on workforce development, making recommendations to Ministries of Health on health care workforce utilization and deployment. Indeed, for 2006, the World Health Day theme was workforce based: Working Together for Health. This is, in part, due to the serious implications of workforce migration from the developing to the developed world but also trying to match the numbers and types of health care workers (skill mix) to country level needs.

Of particular focus in this paper is the contributions made by nursing professionals. The workforce migration issues are most acute in nursing, in part due to societal and economic conditions in many countries of the world. As most nurses in the world are female, gender politics, educational inequalities, and wage disparities are driving forces for migration to countries where wages are higher, there is more public respect for the profession, and nurses have the opportunity for professional advancement [3].

There is widespread recognition that the factors associated with health are multifactorial and include not only health-related risk factors but also social determinants such as poverty, social status, education, employment and other social factors [4]. Thus the purpose of this study was to investigate the relationships of different sets of factors (e.g. social, economic, and health care workforce factors) on HIV/AIDS prevalence rates.

## Methods

Data for the study were derived from a number of WHO and NGO sources. Human resources for health were derived from the Global Health Atlas of Health Workforce 2004 [5]. Health and mortality indicators were obtained from the WHO Statistical Information System (WHOSIS) 2000 [6]. Literacy indicators were collected from UN Fund for Development and Population Assistance (UNFDPA) 2000 [7]. The presence of regulatory bodies for nursing and nursing associations was provided by the International Council of Nurses 2004. Regulation for nursing practice, nursing posts in Ministries of Health and schooling required to become a registered nurse (RN) were obtained from "Nursing in the World" 4th ed., 2000 [8]. HIV/AIDS prevalence rates (2003 estimates) were obtained from the WHO/UNAIDS database [9]. The data from the various data sources were merged into one file at the country level for analysis.

The variables in the data set included the following : 1) number of nurses per 100 000 population; 2) number of midwives per 100 000 population; 3) number of nurses and midwives per 100 000 population; 3) number of physicians per 100 000 population; 4) the presence of nursing regulatory bodies at the national level; 5) the presence of a national nursing association; 6) whether there is regulation for nursing practice; 7) the presence of a WHO Collaborating Centre for nursing (WHO CC); 8) the years of schooling required to become a registered nurse; 9) government expenditure on health as a percentage of total expenditure on health; 10) governmental expenditure on health as a percentage of total general government expenditure; 11) out of pocket expenditure on health as a percentage of private expenditure on health; 12) per capita expenditure on health (USD); 13) per capita expenditure on health in international dollars; 14) private expenditure on health as percentage of total expenditure on health; 15) social security expenditure on health as percentage of general governmental expenditure on health; 16) total expenditure on health as percentage of gross domestic product; 17) total adult literacy rate in %; 18) male adult literacy rate in %; and 19) female adult literacy rate in %. The outcome variable was percentage of adults ages 15 to 49 living with HIV/AIDS, the prevalence rate.

### Analytic method

Two approaches were used for analysis: data mining using classification and regression trees (CART) and standard statistical analyses using ordinary least squares regression. We chose to use both approaches to help us determine, using the data mining approach, which variables were to be used in the standard regression approach. This was particularly important because many of the social and economic variables are multi-collinear. Furthermore, because there was not a specific theoretical model that guided our work (the use of social determinants of health is a philosophical versus theoretical approach), we did not want to exclude variables that may not generally meet the threshold for a stepwise regression approach.

CART is a data mining approach which has been applied successfully in nursing and medical domains. CART can be used for prediction: as an example, CART can help predict levels of HIV/AIDS prevalence rates based on previously learnt data. CART can also be used for interpretative purposes. For example, it can be used to understand better patterns driving specific outcomes, such as HIV/AIDS prevalence rates. In this study, both interpretation and prediction were used.

**Figure 1:**
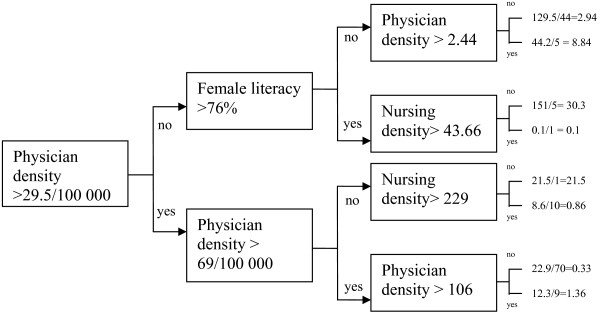
The top level discriminators for HIV/AIDS prevalence.

CART splits data and finds patterns (or sequences of variables explaining consistent outcomes) until all the terminal nodes reach optimum similarity. The selection of the variables is based on maximization of impurity reduction (i.e. variables are selected by the algorithm in the order that best explain the data). The example shown in Figure 1 gives an illustration of patterns of higher incidence of HIV/AIDS prevalence rates using the first three variables to emerge from the CART analysis: physician density, nurse density, and female literacy. The advantage of CART is that new patterns in the data can be discovered.

## Results

### Analysis of patterns

In the first part of the results, authors present a partial description of the key variables that are associated with HIV/AIDS prevalence rates. All variables described under methods were used in developing the CART models. In the next section, authors predict key selected results based on CART to reveal patterns in the data. For this analysis, the CART-derived decision tree and findings show that physician density is the first key discriminator between high and low HIV/AIDS prevalence rates among the 194 countries for which data were collected.

When analysing country profiles from 194 countries, 139 countries had a known level of HIV/AIDS prevalence rate (see Figure 1). The first break was physician density at 29.5 doctors per 100 000 population. For those 54 countries with a physician density < 29.5, the second most important explanatory variable was female literacy (> 76%). For the 49 countries with female literacy < 76%, physician density > 2.44 was the next break variable while for the 6 countries with female literacy > 76%, nursing density was the next break variable.

Physician density greater than 69 was the next break for countries with physician density > 29.5. If physician density was less than 69, then nurse density was the next break for 11 countries. Further to these breaks, it was difficult to draw conclusions because of the small numbers of countries in additional nodes.

The majority of countries (90) had a physician density > 29.5. These countries showed a lower HIV/AIDS prevalence rate compared to countries with physician density <29.5. When physician density was between 29.5 and 69, then nurse density emerged as the second most important criterion associated with lower levels of HIV/AIDS prevalence.

The average HIV/AIDS rate was 1.36 (9 countries) when physician density was greater than 106, which indicated that a very high level of physician density alone is not a good predictor of lower levels of HIV/AIDS prevalence. Thus, a CART-based approach provides a statistically valid and logical explanation of complex predictions including many variables of different types, even qualitative.

### Predictive modelling

One of the advantages of using CART – similar to other benchmarking techniques – is the ability to predict outcomes on the basis of existing clusters or rules classifying the problem domain. For this research, we decided to use CART derived clusters to help identify which variable(s) can best explain HIV/AIDS prevalence rates in selected countries around the world. The four countries selected for this purpose were Botswana, Swaziland, Thailand, and Zimbabwe. These four countries were selected on the basis of 1) high levels of HIV/AIDS prevalence rates and 2) the presence of data for the potential explanatory variables. The selection of a different set of countries would not have likely affected the results. The first step was to eliminate them from the sample, one by one. The second step considered recalculating new cluster values. The third step assessed whether the nodes and their associated values had changed, and if so, by how much. In order to perform this analysis, authors compared each country profile in the new cluster tree and assessed the variation between predicted values and actual values of HIV/AIDS prevalence rates.

CART produced very accurate (greater than 95% accuracy) outcomes using the methods described in Table 1. In the case of Swaziland, the accuracy rose to 100%. Smith, Scherer and Hauser [10] argued that when applying CART, a 97.2% accuracy level should be considered as "effective".

**Table 1:** Accuracy of CART for predicting HIV/AIDS prevalence rates

	Predicted HIV/AIDS prevalence rates	Actual HIV/AIDS prevalence rates (% of adults ages 15 to 49 with HIV)	Δ	Female literacy (%)	Physician density (# per 100 000)	Nurse density (# per 100 000)
Botswana	38.8	37.3	-1.5	79.8	28.76	241.08
Swaziland	38.8	38.8	0	78.6	17.62	320.32
Zimbabwe	28.9	24.6	-4.3	84.6	5.73	54.16
Thailand	0.6	1.5	0.9	93.9	30.08	161.7
Σ	107.1	102.2	-4.9			

### Standard multiple regression analyses

Standard ordinary least squares regression was performed using some of the same variables used in the CART approach, with the intent to build the best explanatory model (highest explained variance and theoretically important independent variables). Because of non-normal distribution of HIV/AIDS prevalence rates, the log of the HIV/AIDS prevalence rates was used. The statistical assumptions for multiple regression were examined and met. Because there was multiple co-linearity for some of the independent variables, particularly the socio-economic ones, the final model contains two independent variables (physician density and nurse density) and one covariate (total per capita expenditure on health in international dollars). The final model was significant (F = 28.0, p < .001) with 36% explained variance (adj R^2 ^= .36). The workforce variables, when controlling for the per capita expenditure on health, were both significant: physician density with standardized regression coefficients (beta) of -0.52 and nurse density = -0.22. Most simply, this is the change in the log percentage of adults ages 15 to 49 with HIV for a unit change in the independent variable. The interpretation, therefore, is that even when controlling for per capita expenditures on health, both physician and nurse density make a contribution to HIV/AIDS prevalence rates for those between 15 to 49 years of age. When controlling for physician density, nurse density had an independent association with HIV/AIDS prevalence rates for the 15 to 49 age group. Of note, per capita expenditure on health in international dollars had less relative influence on prevalence rates than either of the workforce variables (beta = 0.17, p = 0.04) (See Table 2).

**Table 2:** Regression results from OLS regression (N = 144)

	Unstandardized regression coefficient	Standardized regression coefficient	t value	p value
Physician density per 100 k	-.006	-.52	5.55	<.001
Nurse density per 100 k	-.001	-.22	2.31	.02
Per capita total expenditures on health in international $	.000	.17	2.04	.04

Adj R^2 ^= 0.36; F = 28.0, p < .001

## Discussion

This paper describes how a data mining approach and standard statistical analyses were able to support the link between health workforce composition and HIV/AIDS prevalence rates in selected countries. In summary, nursing and physician density were both associated with HIV/AIDS prevalence rates. The other important variable was female literacy level. There were a number of variables that did not emerge in the data mining analysis that have been conventionally used to explain HIV/AIDS prevalence, in particular socioeconomic status variables, such as expenditures on health and gross domestic product.

There are a number of explanations for our findings. Consistent with the emphasis on the social determinants of health, there is evidence that higher overall educational attainment for both genders was associated with lower prevalence rates of HIV/AIDS in Uganda [11]. Similar trends were identified in multi-country reviews where HIV/AIDS prevalence rates fell among the more educated segments of the population, likely in response to educational campaigns related to safer sex practices [12]. Thus, our findings on female literacy are consistent with the findings from other research and consistent with Marmot's contention that improvement in health requires focus on more than just the health sector.

In countries where HIV/AIDS over-stressed the health care system and where pharmaceutical treatments are not available or are not widely available, home-based care, organized and delivered by nurses, may be the only available form of health care. Thus an investment in home care nursing in these countries can reduce the spread of HIV/AIDS through community and public health nursing approaches [13].

To lessen the impact of HIV/AIDS on health care systems globally will require a multi-faceted approach. It may not be sufficient to simply request more financial support without revising current approaches to train and retain nurses and physicians and to increase female literacy. Governments may want to consider programs specifically targeted to strengthen and alternatively distribute the nursing workforce given that our findings showed nurse density having an association with HIV/AIDS prevalence rates. Strengthening the nursing workforce may also require additional investments in education to prepare additional number of nurses where there is an absolute shortage of personnel. Any such programs will also need to consider improving the working conditions of nurses to ensure that they do not fall victims to the disease they help to prevent through occupational exposure. Concurrently, there will have to be multi-sectoral efforts to improve the status of women through education. A recent paper identified "economic, social, and political empowerment of women" as a key necessary driver behind new policies on HIV/AIDS [14]. Our data did confirm the impact of female literacy in combination with nurse density on positive HIV/AIDS outcomes. Thus continued efforts on increasing female literacy through investment and the development of strategies to promote the schooling of girls may be helpful in addressing the HIV/AIDS pandemic [15].

There are areas for further research which will have to be addressed. Data obtained for nurses in this study could not differentiate between the various educational background and practice of nurses worldwide. Had we been able to run a more detailed analysis aided by accurate description of the nursing workforce, results would have allowed for more precision concerning the impact on HIV/AIDS outcomes. Another limitation was the lack of ability to separate the number of midwives and their contributions to preventing HIV/AIDS from the nursing data. There are large variations in practice; in some countries midwives have their independent practice, in other countries nurses who receive midwifery training do both, while a third scenario is where all midwives must be trained nurses. We acknowledge that there is an increased need to have trained HIV/AIDS health care providers, especially in areas where resources are scarce [16]. Yet we did not have data on the degree or extent of HIV/AIDS training among health care providers included in the present study. Finally, current HIV/AIDS prevalence is in large part a reflection of historical workforce composition. A cross-sectional examination as presented here has limitations between data of different ages and the temporal and complex nature of health for persons with HIV/AIDS, whereby the availability of ARVs and the workforce interact with social determinants of health like HIV/AIDS prevalence.

In addition, further country-level information on how nursing is integrated within the culture and within the health care system of that particular country may be informative in identifying best nursing workforce practices for health care structure, licensure/registration, scope of practice, education and support of nursing education, collaboration with physicians, and the use of traditional or community health care workers. New models are needed to assess HIV/AIDS testing, care, and treatment impact on patients and their communities, all of which are largely influenced by culture and setting.

There are a number of limitations to our study. First, we were using country level data and there are some countries where prevalence reports are not made public. The inclusion of these countries would have benefited this research. Second, by the use of a reduced dataset, we risked over-simplifying complex political and health care system characteristics that could have modified results. Third, because HIV/AIDS is often associated with stigma in many parts of the world, we may have overlooked the effect of such factors in the absence of accurate data. Fourth, because nursing workforce educational levels widely varied, we could not distinguish the contributions made by the various levels of nurses. Fifth, the data sources were from different years and different sources of information, which may have resulted in missing some of the dynamic nature of the change in the variables used in the combined data base. Finally, our study variables indicate correlations and we are not implying cause-and-effect relationships.

## Conclusion

Looking at our outcomes, it is evident that nursing density has a significant association with HIV/AIDS outcomes in many countries. Investment in the nursing workforce by creating and supporting additional nursing education programs may have a positive impact on HIV/AIDS prevalence rates in many developing nations. In addition to the preparation of new nurses and midwives, increasing satisfaction of the currently employed and improving their working conditions may decrease nurse migration [3]. Work by WHO and NGOs in influencing health policy to assist governmental agencies to better utilize nurses and midwives' unique contributions to reducing HIV/AIDS infections may also have an impact upon MDG achievements. These policies should walk hand-in-hand with work by NGOs to support and assist governments to increase female literacy that may have a pay-off in lower HIV/AIDS prevalence rates.

Attainment of the MDG for HIV/AIDS will not be achieved by opening up pharmaceutical treatment in the form of ARVs without a commensurate investment in the health care workforce which provides the necessary care for patients living with HIV/AIDS. Rightfully, much international attention is currently focused on the unequal access to pharmaceutical treatment for HIV/AIDS patients. But even if the drugs were widely available at low cost tomorrow, the current, insufficient number of health personnel would not be enough to match the millions of infected individuals waiting for the attention of health care worker.

## Competing interests

The authors have no competing financial interests. Dr Zrinyi was formerly employed by the WHO but has no current financial or other ties to the WHO.

## Authors' contributions

MZ developed the merged data set. OLC performed the data mining. EAM performed the multiple regression analysis. The generation of the idea and writing of the paper was a three way effort in drafting and revising the final copy. All three authors approved the final version.
